# Vascular tissue reconstruction by monocyte subpopulations on small-diameter acellular grafts via integrin activation

**DOI:** 10.1016/j.mtbio.2023.100847

**Published:** 2023-10-28

**Authors:** Atsushi Mahara, Manabu Shirai, Raghav Soni, Hue Thi Le, Kaito Shimizu, Yoshiaki Hirano, Tetsuji Yamaoka

**Affiliations:** a Department of Biomedical Engineering, National Cerebral and Cardiovascular Center Research Institute, Kishibe Shimmachi, Suita Osaka, 564-8565, Japan; b Omics Research Center, National Cerebral and Cardiovascular Center Research Institute, Kishibe Shimmachi, Suita Osaka, 564-8565, Japan; c Faculty of Chemistry, Materials and Bioengineering, Kansai University, 3-3-35 Yamatecho, Suita, Osaka, 565-8680, Japan

**Keywords:** Tissue engineered vascular graft, Monocyte, Integrin, Regeneration

## Abstract

Although the clinical application of cell-free tissue-engineered vascular grafts (TEVGs) has been proposed, vascular tissue regeneration mechanisms have not been fully clarified. Here, we report that monocyte subpopulations reconstruct vascular-like tissues through integrin signaling. An Arg-Glu-Asp-Val peptide-modified acellular long-bypass graft was used as the TEVG, and tissue regeneration in the graft was evaluated using a cardiopulmonary pump system and porcine transplantation model. In 1 day, the luminal surface of the graft was covered with cells that expressed CD163, CD14, and CD16, which represented the monocyte subpopulation, and they exhibited proliferative and migratory abilities. RNA sequencing showed that captured cells had an immune-related phenotype similar to that of monocytes and strongly expressed cell adhesion-related genes. In vitro angiogenesis assay showed that tube formation of the captured cells occurred via integrin signal activation. After medium- and long-term graft transplantation, the captured cells infiltrated the tunica media layer and constructed vascular with a CD31/CD105-positive layer and an αSMA-positive structure after 3 months. This finding, including multiple early-time observations provides clear evidence that blood-circulating monocytes are directly involved in vascular remodeling.

## Graphical abstract

**Figure:**
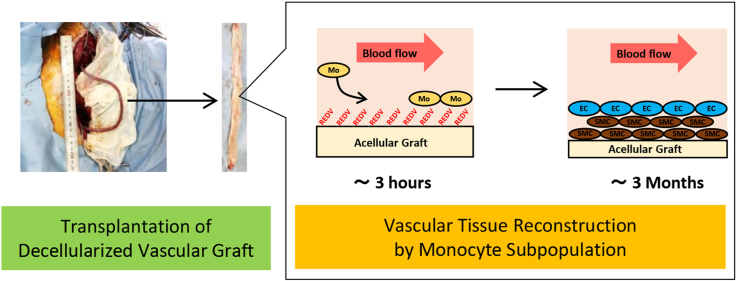

## Abbreviations

TEVG: tissue-engineered vascular graft
EPC: endothelial progenitor cell
ECFC: endothelial colony-forming cell
REDV: peptide Arg-Glu-Asp-Va
POG7G3REDV: sequence (Pro-Hyp-Gly)7-Gly-Gly-Gly-Arg-Glu-Asp-Val
CLSM: confocal laser scanning microscopy
ACT: activated coagulation time
SEM: scanning electron microscopy
RCA: right carotid artery
HE: hematoxylin and eosin
EVG: Elastica van Gieson
vWF: von Willebrand factor
AMR: Applied Medical Research Laboratory
pEC: porcine endothelial cell
EBM: endothelial basal medium
PBS: phosphate buffer saline
FSC: forward scatter
SSC: side scatter

## 1 Introduction

Decellularized vascular grafts have great potential for realizing small-diameter artificial vascular grafts with an inner diameter of 4 mm or less; however, they are not currently approved for clinical applications. In the case of the decellularized tissue-engineered vascular grafts (TEVGs), vascular tissues must be reconstructed by the action of host cells on the graft. Endothelial progenitor cells (EPCs) have been cited as an appropriate cell source for reconstructing vascular tissues for cell-free TEVGs [[1], [2], [3]]. The therapeutic efficacy of EPCs for limb ischemia has been investigated in clinical trials, and favorable clinical trends have been reported [4]. Moreover, current scientific evidence based on cell biology suggests the presence of two different cell populations in mononuclear cells: early and late EPCs [5]. Some groups suggested that early EPCs can promote endothelial cell formation via paracrine effects, while late EPCs are known as endothelial colony-forming cells (ECFCs). Although late EPCs contribute to vascular repair and blood vessel formation, early EPCs are not [6,7]. However, a consensus has not been reached regarding the existence of EPCs [8].

Recently, the molecular mechanisms of angiogenesis and vasculogenesis have been elucidated based on tissue-resident stem/progenitor cells [9]. Tissue-resident ECFCs have been identified in various human tissues, such as adipose [10] and placenta tissue [11]. Tissue-resident endothelial stem cells have also been identified in mouse blood vessels, adipose tissue, and skin tissue [[12], [13], [14]]. McDonald et al. demonstrated that an 0.8-mm long vascular injury site in the descending aorta of mice was regenerated within 3 days by distinct cell populations arising from differentiated endothelial cells but not circulating cells [15]. Okuno et al. reported that bone marrow-derived cells contribute to wound healing as macrophages while vascular cells did not^8^.

However, considering realistic scenarios and clinical situations, the graft length should be more than 1 cm. Moreover, endothelialization is accomplished only in a *trans*-anastomotic manner, which may seem counterintuitive. Both human clinical trials and large animal studies have indicated that circulating ECFC are involved in tissue regeneration [16]; however, these cells have not been isolated as a single-cell population [9]. VEGF- and SDF-1-modified grafts improved graft patency by rapid endothelialization supported by monocytes and macrophages [17]. Smith Jr. et al. recently reported that endothelialization of a 50-cm long graft was accomplished without cellular ingrowth from the anastomotic site in a large-animal model and blood circulating monocytes contributed to endothelial regeneration [17]. Therefore, the tissue regeneration process depends on the injury size, and regeneration might be achieved not only by resident cells but also by circulating cells [18]. However, limited data are available on vascular tissue regeneration by blood-circulating cells and previous studies have not clearly demonstrated the mechanism or time scale in detail.

**Fig. 1:**
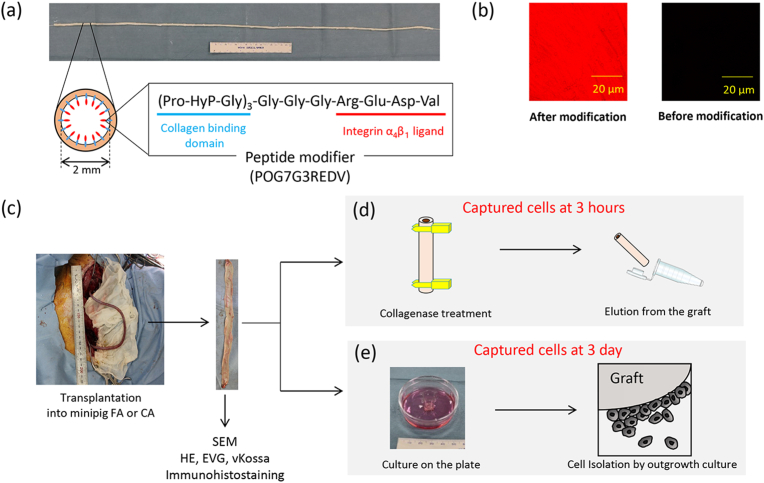
**Isolation process of captured cells on the REDV-modified decellularized surface. a**, Gross appearance and schematic images of peptide-modified acellular graft. **b,** Luminal surface of the graft before and after peptide modification observed by conforcal laser scanning microscopy (CLSM). Carboxytetramethylrhodamine-conjugated REDV peptide was used in this experiment. **c-e**, Experimental scheme for the isolation of captured cells. For histological evaluation, grafts that had been transplanted for more than 1 day using femoral artery bypass were evaluated. The captured cells were isolated (**d**) 3 h and (**e**) 3 days after transplantation to carotid artery and femoral artery bypass, respectively.

Here, we investigated vascular tissue regeneration at both early time points and after long-term periods using TEVGs of clinically useful size. The monocyte subpopulation was shown to infiltrate the medium layer of the graft after matrix degradation, and it was directly involved in vascular remodeling of not only the intima but also the medial region. In a previous study, we developed peptide-modified acellular grafts (Fig. 1a) [[19], [20], [21], [22]] and modified the surface with the integrin α4β1 ligand peptide Arg-Glu-Asp-Va (REDV). We demonstrated the patency of a long-bypass graft with an inner diameter of 2 mm and length of 20–30 cm using a minipig femoral-femoral bypass model [22]. The grafts maintained a stable blood flow without any thrombogenic formation, and the luminal surface was covered with cells at 7 days after transplantation, even at approximately 15 cm from the anastomotic site. We also reported that peptide modification of the graft effectively suppressed the initial deposition of fibrin clots [20,23,24]. These results imply that circulating blood cells mainly contribute to vascular reconstruction. In this study, we clearly identified captured cells on the luminal surface of the graft in an early period of 1–3 h after transplantation using a blood-circulating system with a cardiopulmonary bypass system. Moreover, we demonstrate that the monocyte subpopulation reconstructs vascular tissues with endothelium and tunica media layers within 3 months of transplantation. This presents spatiotemporal evidence that the circulating monocyte directly regenerates the vascular tissue in the adult body.

## 2 Material and methods

### 2.1 Peptide-modified decellularized graft

This study used peptide-modified decellularized grafts (Fig. 1a) prepared from an ostrich carotid artery as previously reported [22]. Briefly, isolated carotid arteries were decellularized via the ultrahigh hydrostatic pressure method applied using a cold isostatic pressurization machine (Dr. Chef; Kobelco, Kobe, Japan), which contained a pressure transmission fluid consisting of ethylene glycol and water. The pressure was increased to 980 MPa at a rate of 65.3 MPa/min and then maintained within the chamber for an additional 10 min. The specimen was washed with 40 U/mL DNase I (Roche Applied Science, Indianapolis, IN, USA), 20 mM MgCl_2_, and antibiotics for 3 days at 37 °C. A custom-synthesized peptide with the sequence (Pro-Hyp-Gly)_7_-Gly-Gly-Gly-Arg-Glu-Asp-Val (POG7G3REDV), where Hyp stands for hydroxyproline, was purchased from Scrum Inc. (Tokyo, Japan). Decellularized carotid arteries were immersed in a 10 μM peptide solution with saline and incubated at 60 °C for 1 h, and then the graft was cooled to 25 °C for 1 h. Peptide-modified and unmodified grafts are denoted P-graft and Un-graft, respectively. The grafts with an inner diameter of 2 mm were used.

To confirm a uniform modification with the peptide, carboxytetramethylrhodamine-conjugated POG7G3REDV was used. The images were acquired using the FV1000-D CLSM system (Olympus, Tokyo, Japan) (Fig. 1b).

### 2.2 Blood response to the graft

All animal experiments were conducted in accordance with the Guidelines for Animal Experiments established by the Ministry of Health, Labor, and Welfare of Japan and the National Cerebral and Cardiovascular Center Research Institute in Japan. The protocol was approved by the Committee on the Ethics of Animal Experiments of the National Cerebral and Cardiovascular Center Research Institute (Permit Number:009017). Göttingen minipigs, purchased from Ellegaard Göttingen Minipigs A/S (Dalmose, Denmark), were anesthetized with intravenous injection of 100 mg/h 1 % propofol (Diprivan; AstraZeneca, Wilmington, DE).

For a 1-h evaluation, we used an artificial cardiopulmonary system that allows blood circulation to be started without a vascular suturing process to control precise blood circulation time. To evaluate many samples with as few animals as possible according to the 3Rs principles in animal experimentation, blood circulations for 3 h and more than 1 day were evaluated by graft transplantation to the carotid artery and femoral-femoral bypass position, respectively.

#### 2.2.1 Ex vivo blood response

An artificial cardiopulmonary system (Inspire 8 oxygenator, LivaNova, Mirandola, Italy) was used to evaluate thrombus formation on unmodified and peptide-modified grafts during 1 h of blood circulation (Supplemental Fig. S1). Activated coagulation time, monitored by activated coagulation time (ACT) (Medtronic, Inc., Minneapolis, MN), was controlled at approximately 400 s during the experimental surgery by heparin injection (Novo-heparin; Fuso Pharmaceutical Industries, Osaka, Japan). The artificial cardiopulmonary system was attached to the minipig at the right ventricle and ascending aorta for blood removal and resupply via cannula, respectively. The P-graft and Un-graft were connected to the blood removal and sending cannula lines, and the luminal surface of these grafts was exposed to blood flow. After that, the luminal surface was washed with saline and fixed with 4 % glutaraldehyde. The specimen was dehydrated with ethanol, immersed in *tert*-butyl alcohol, and dried under vacuum. The luminal surface was coated with a gold layer in an ion coater (IB-3 ion-coater; Eiko Engineering, Ibaraki, Japan) and observed using scanning electron microscopy (SEM) (JCM 5700 microscope JEOL, Tokyo, Japan).

#### 2.2.2 In vivo blood response

Cell capture over 3 h was evaluated by temporarily replacing the right carotid artery (RCA) with a graft with a length of approximately 20 cm (Fig. 1c, Supplemental Fig. S2). The graft was connected to the carotid artery in an end-to-end fashion by 8-0 Prolene sutures. The ACT was controlled at approximately 200 s during graft transplantation.

To evaluate cell capture and tissue regeneration for more than 1 day, femoral-femoral crossover bypass methods were used with a 20-cm long P-graft (Fig. 1c). For histological evaluation, the graft was transplanted for 1 day, 3 days, 1 week, 3 weeks, 5 weeks, and 3 months with the bypass model. ACT was controlled in the same way as carotid artery transplantation. The left and right femoral arteries were exposed and clamped using disposable microvascular clamps (Bear Medic Co., Ibaraki, Japan). The graft was connected to the left femoral artery in a side-to-end fashion and to the right femoral artery in an end-to-end fashion using 8-0 Prolene. Blood flow was checked using echocardiography (Prosound α7; Hitachi-Aloka Medical, Tokyo, Japan), laser-Doppler flowmetry (Omega Flow FLO-C1; Omegawave, Tokyo, Japan), and an electromagnetic blood flow meter (Transonic TS420, Transonic, Ithaca, NY). Additional anticoagulation medications were not administered after transplantation.

The grafts were removed from the surgically implanted position and then fixed with 10 % formalin neutral buffer solution and 4 % glutaraldehyde for histological and SEM observation. Histological staining by hematoxylin and eosin (HE), Elastica van Gieson (EVG), von Willebrand factor (vWF), and von Kossa was carried out by the Applied Medical Research Laboratory (AMR; Osaka, Japan). Immunostaining was also carried out using anti-CD31 (MCA1746, Bio-Rad Laboratories, Montreal, Quebec), CD34 (bs-0646R, Bioss Antibody Inc., Boston, MA), CD105 (bs-0579R, Bioss Antibody Inc.), and Flk-1 antibodies (bs-0565R, Bioss antibody Inc.). Table 1 summarizes the types and roles of the antibodies used in this study.

**Table 1:** The surface maker for the antibody used in this study.

Category	Antigen	Expressing cell type
Monocyte subset marker	CD14	Mo/MΦ
	CD16	Mo/MΦ/NK/GL
	CD163	Mo/MΦ/Porcine Mo specific marker
Endothelial-related marker	CD31	EC/HPC
	CD105	EC/Mo
	CD34	EPC/HPC
	Flk-1	EC/EPC

Mo: Monocyte, NK: Natural killer cell, MΦ: Macrophage, GL: Granulocyte.

EC: Endothelial cell, HPC: Hematopoietic progenitor cell, EPC: Endothelial progenitor cell.

### 2.3 Isolation of captured cells from the transplanted graft

The cells captured on the graft after RCA transplantation for 3 h were isolated using collagenase treatment (Fig. 1d). The luminal surface of the graft was coated with a 0.2 % collagenase solution (WAKO Chemicals, Tokyo, Japan), and then incubation was performed for 10 min at 25 °C. The solution was collected in a 15 mL centrifuge tube, and the graft was washed three times with the cell culture medium. The cells were then collected by centrifugation at 100×*g* for 3 min, and the cell pellet was used for subsequent surface marker and RNA sequencing analyses. Porcine endothelial cells (pEC) were isolated by the same procedure from normal porcine aortas.

### 2.4 Cell culture and isolation

The graft was isolated 3 days after transplantation. Squared grafts measuring 1 cm × 1 cm were prepared, and the luminal surface was placed in contact with a polystyrene cell culture plate (Iwaki, Tokyo, Japan) (Fig. 1e). The plate was incubated with endothelial basal medium (EBM-2; Lonza, ASwitzerland) supplemented with EGM-2 MV growth factor kit (Lonza, Switzerland) under cell culture conditions. After 3 days, the tissues were removed from the culture dish and the outgrowth cells were cultured. Two cell types were isolated using single-colony isolation with a limiting dilution method. Briefly, the cell suspension was diluted and cultured in a 96-well plate under the condition that one or fewer cells were seeded in one well. The growing colonies were separated, and morphologically different cells with filopodia and spindles were named CC-3D_1_ and CC-3D_2_, respectively.

Human umbilical vein endothelial cells (ECs, KE4109, Kurabo, Biomedical Business, Japan) and pECs were cultured on a polystyrene surface using EBM-2 supplemented with EGM-2 and a growth factor kit (Lonza, Switzerland). Fibroblast cells (FCs, JCRB0615, NIH/3T3 clone5611) were cultured in Dulbecco's modified Eagle's medium (low glucose, Gibco) supplemented with 10 % fetal bovine albumin and antibiotics. The cells were grown to confluence. The cultures were maintained in a humidified atmosphere containing 95 % air and 5 % CO_2_ at 37 °C. The culture medium was changed every 2 days, and the cells typically reached confluence within 3–4 days. After the cells reached confluence, they were washed with 37 °C phosphate buffer saline (PBS, pH = 7.4, Gibco, Waltham, USA) and sub-cultured using 0.05 % trypsin/0.01 % EDTA (Gibco, Waltham, USA).

Porcine mononuclear cells were isolated using Ficoll density gradient centrifugation. Blood (10 mL) was aspirated from the porcine aorta and diluted with an equal volume of PBS. A sample of diluted blood (4 mL) was placed in a 15 mL centrifuge tube and topped with 3 mL of Ficoll-Paque media (Ficoll-Paque PREMIUM [Density 1.084], GE Healthcare Bio-Sciences AB, Sweden). The tube was centrifuged with 400×*g* at 20 °C for 30 min. The mononuclear layer was aspirated with a needle, and the cells were suspended in 6 mL of PBS. Subsequently, the cell suspension was centrifuged at 100×*g* for 10 min, and the platelet supernatant was excluded. The cell pellet was suspended in the buffer and used in the next experiments.

### 2.5 Surface marker characterization and monocyte subpopulations cell sorting

Cell surface marker expression was evaluated by FACSCalibur flow cytometry (BD Biosciences, San Jose, CA) and Guava EasyCyte flow cytometer (Merck Millipore, USA). The types and roles of the antibodies used in this study are summarized in Table 1. The cells were stained with PE-conjugated anti-CD31 antibody (MCA1746PET, Bio-Rad Laboratories, Montreal, Quebec), anti-CD34 antibody (bs-0646R-PE, Bioss Antibody Inc.), anti-CD105 antibody (bs-0579R-PE, Bioss Antibody Inc.), and anti-Flk-1 antibody (bs-0565R-PE, Bioss Antibody Inc), anti-CD163 antibody (bs-2527R-PE, Bioss Antibody Inc.), anti-CD14 antibody (MCA1218F, Bio-Rad Laboratories, Inc., Hercules, CA), and anti-CD16 antibody (MCA1971PE, Bio-Rad Laboratories, Inc.). The cells were suspended into 2 % FBS/PBS and mixed with the antibodies. The solution was incubated for 30 min at 4 °C, and the cells were cooled on ice until use.

Immunostained cells were also evaluated using CLSM. The cultured cells on the glass bottom dish were fixed with 3 % formaldehyde solution in PBS at 4 °C for 20 min. After washing the cells with PBS, antibody solution was added and the cells were incubated at 25 °C for 1 h. The antibody solution was prepared according to the following protocol: After incubation, the cells were washed with PBS. DAPI in PBS (10 μg/mL) was added to the cells, which were observed using a FV1000-D CLSM system (Olympus, Tokyo, Japan). LDL uptake was also evaluated using Dil-ac-LDL (BT-902, Biomedical Tech, Inc., Stoughton, Massachusetts, USA). Dil-ac-LDL (10 μg/mL) was added to cultured cells and incubated at 37 °C for 4 h. After washing with PBS, the cells were fixed with 3 % formaldehyde solution at 25 °C for 20 min. The cells were washed with PBS, and then DAPI (2 μg/mL) was added. The cells were subsequently observed using CLSM.

Monocyte subpopulations were isolated from the mononuclear cells using BD FACSAria Fusion cell sorter (BD Biosciences, San Jose, CA). For cell sorting, 4 × 10^6^ cells were labeled with anti-CD14, anti-CD16, and atni-CD163 antibodies as described above and resuspended in 2 % FBS/PBS. The monocyte subpopulation was divided into two populations using these antibodies; monocyte populations with CD14Low/CD16Low/CD163+ and CD14+/CD16+/CD163+ are hereafter denoted MoN and MoP, respectively.

### 2.6 RNA sequencing

Total RNA was extracted from CC-3H, CC-3D_1_, CC-3D_2_, MoP, MoN, and pEC cells using an miRNeasy-micro/mini kit (Qiagen, Hidden, Germany) and treated with RNase-free DNase I (Qiagen). The RNA integrity number was measured using an Agilent 4200 TapeStation (Agilent, Santa Clara, CA, USA) to assess the quality of the isolated total RNA. mRNA sequencing libraries from high-quality total RNA were prepared using a TruSeq stranded mRNA library prep kit (Illumina, San Diego, USA). After evaluating the library quality, sequencing was performed with 75 bp paired-end reads using the NextSeq 500 High Output Kit (Illumina). After demultiplexing with Bcl2fastq (Illumina), the quality of the raw reads was checked, trimmed, and aligned to the minipig (Sus scrofa) reference genome (Sscrofa 11.1) on the CLC genomics workbench ver. 20.0 (Qiagen). K-means, functional annotation, and pathway enrichment analyses were performed with iDEP 0.96 (http://bioinformatics.sdstate.edu/idep96/).

### 2.7 Tube formation assay

Endothelial function was evaluated by a tube formation assay using the Endothelial Tube Formation Assay kit (CBA-200, Cell Biolabs, Inc., San Diego, CA, USA). The assay was performed in accordance with the manufacturer's instructions. Briefly, 40 μL of pre-chilled Matrigel were added to 96 well microplates (Iwaki Glass, Tokyo, Japan) and the plate was incubated at 37 °C for 1 h. ECs, CC-3D_1_ and CC-3D_2_ (2 × 10^4^ cells) were seeded on the Matrigel surface and incubated for 6 h. After incubation, the cell morphology was observed by a microscope (Eclipse TE-300, NIKON, Tokyo, Japan) equipped with a digital camera (Floyd, Wraymer Inc., Osaka, Japan). Tube formation was analyzed using ImageJ software installed with an angiogenesis analyzer [25]. Y27632, which was purchased from FUJIFILM Wako Pure Chemical (Kyoto, Japan), was used as the ATP-competitive ROCK inhibitor. The morphology of the OCs was evaluated by phalloidin staining. The cells were fixed with 3.7 % formaldehyde solution for 10 min at room temperature and then immersed in 0.1 % Triton X-100 in PBS for 5 min. Fixed cells were stained with rhodamine-phalloidin (Life Technologies, Grand Island, NY, USA) and DAPI solution (Dojin Chemical Co., Kumamoto, Japan). After staining, the specimens were observed using CLSM.

### 2.8 In vitro cell differentiation assay

Blood was collected from the tail artery of the minipigs, and then RBC lysis was performed using 1 × RBC lysis buffer (pluriSelect Life Science, Germany) to obtain a leukocyte suspension. Briefly, 5 mL of whole blood was mixed with 45 mL of lysis buffer, incubated at room temperature for 15 min, and centrifuged at 300×*g* for 10 min to remove lysis buffer. The leukocytes were washed with PBS and resuspended in cell medium **(**Porcine EC Growth Medium Kit, Cell Application Inc., USA). The P-graft and Un-graft (length 15 mm) were cut along the longitudinal axis and placed on 4 chamber slides (AGC Techno Glass, Japan). The graft lumen surface was maintained on the upper side and held using sterile needles to avoid unnecessary folding in the chamber. Leukocyte suspension (1 ml, 2.5 × 10^5^ cells) was added to each chamber and incubated (cell IQ CO2 incubator, Panasonic, Japan) for 7 and 14 days, respectively. The cell medium was carefully changed every 5 days. The surface marker expression of cells on the incubated graft was evaluated using CLSM (Olympus, Tokyo, Japan) and flow cytometry. The cells were stained with FITC-conjugated CD14 (MCA1218F), PE-conjugated anti-CD31 (MCA1746PET), PE-conjugated CD16 (MCA1971PE), supplied by Bio-Rad Laboratories, Montreal, Quebec, and CD34 (bs-0646R-PE, Bioss antibody Inc., Boston, MA, USA). In brief, incubated grafts were washed with PBS, cells on the lumen surface were fixed with 5 % formaldehyde solution at 25 °C for 20 min. Then, 0.1 % Triton X-100 was added at room temperature for 5 min. The graft was washed with PBS, and the staining antibody was added and incubated at 4 °C for overnight. The stained grafts were observed using CLSM. The surface marker expression levels were quantified using flow cytometry. The cells on the lumen surface were collected by trypsinization (trypsin/EDTA solution, Lonza, USA) and resuspended in PBS for 30 min at 4 °C with antibodies against surface markers CD14, CD16, CD31, and CD34. Cell labelling was performed according to the manufacturer's instructions.

### 2.9 Statistical analysis

Quantitative data were expressed as the mean ± standard deviation. One-way analysis of variance followed by Tukey's honest significant difference post-hoc test were used for parametric analyses.

## 3 Results

### 3.1 Blood response to the graft surface

**Fig. 2:**
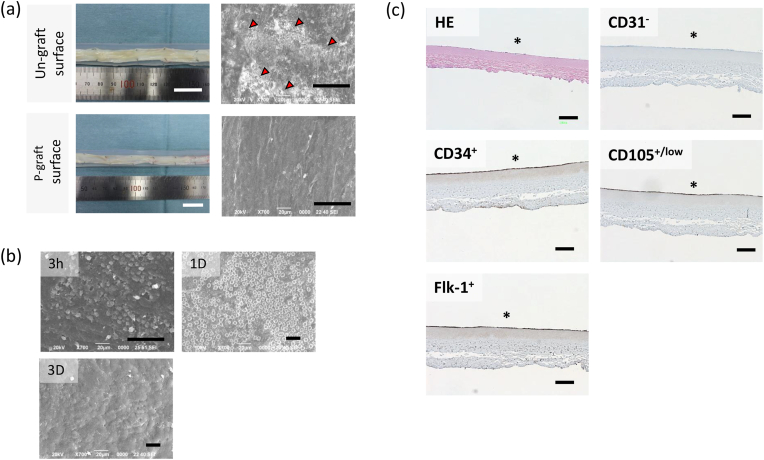
**Blood response to the luminal surface of the P-graft and Un-graft. a,** Gross images and SEM observation of the luminal surface of the P-graft and Un-graft. Blood was circulated for 1 h by a cardiopulmonary pump system. The red arrowhead indicates clot deposition. **b**, SEM images of the luminal surface of the P-graft after transplantation for 3 h, 1 day, and 3 days. **c**, Cross-section images of the P-graft after transplantation for 1 day. The sections were stained with HE, anti-CD31, anti-CD34, anti-CD105, and anti-Flk-1 antibodies. **Asterisks indicate the luminal side.** (For interpretation of the references to color in this figure legend, the reader is referred to the Web version of this article.)

After exposing the graft surface to blood flow for 1 h using an artificial cardiopulmonary pump system (Supplemental Fig. S1), the luminal surface was observed. Although the gross images were nearly equivalent (Fig. 2a), the SEM images revealed microthrombus formation on the Un-graft surface. In contrast, no clot deposition was observed on the surface of the P-graft. When the P-graft was transplanted for 1 and 3 days, almost all of the luminal surface was covered with cells at 1 day and layered structures were formed on the luminal surface at 3 days (Fig. 2b). Immunohistostaining of the P-graft was performed after 3 days, and it showed that cells expressing CD34, CD105, and Flk-1 (but not CD31) accumulated as a single cell layer (Fig. 2c). These results indicated that the REDV peptide on the luminal surface of the P-graft captured blood-circulating cells in just 1 day and induced cell layer formation rapidly without thrombosis formation. Conversely, the luminal surface of the Un-graft was rapidly covered with the thrombus. We previously reported that the Un-graft becomes occluded after graft transplantation [22]. Considering that our data indicated that the cells were not captured on the Un-graft surface, the captured cells on the P-graft were evaluated in the following experimental section.

### 3.2 Flow cytometry analysis of captured cells at 3 h after blood circulation

**Fig. 3:**
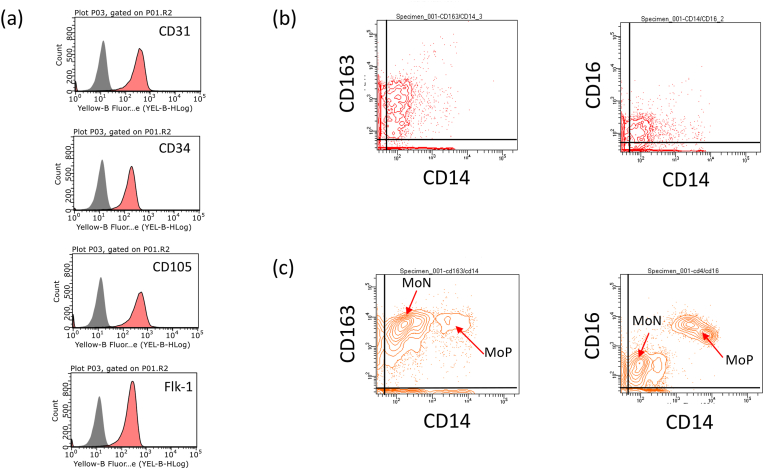
**Surface marker analysis of captured cells and porcine monocyte.** The captured cells were isolated from the 3 h transplantation P-graft. **a**, Expression levels of CD31, CD34, CD105 and Flk-1 in CC-3H were indicated. **b**, Two-dimensional expression patterns of CD163/CD14 and CD16/CD14 in the captured cells were plotted. **c**, Two-dimensional expression patterns of CD163/CD14 and CD16/CD14 in the porcine monocyte were plotted. The MoN and MoP populations were expressed CD14^Low^/CD16^Low^/CD163^+^ and CD14 + /CD16 + /CD163 + , respectively.

To characterize the captured cells isolated from the P-graft after RCA transplantation for 3 h, the surface markers of the CC-3H cells were analyzed by flow cytometry. The captured cells expressed CD31, CD34, CD105, and Flk-1 (Fig. 3a), and they also expressed the porcine monocyte marker CD163 [26] within the low CD14 expression fraction (Fig. 3b). Two-dimensional analysis of the CD14/CD16 and CD14/CD163 expression data suggested that CC-3H expressed CD14^Low^, CD16^Low^, and CD163^+^. The expression patterns of CD14, CD16, and CD163 in porcine monocytes are shown in Fig. 3c. Porcine monocytes had two cell populations in the two-dimensional map of CD14/CD16 and CD14/CD163 expression. The monocyte populations with CD14^Low^/CD16^Low^/CD163^+^ and CD14^+^/CD16^+^/CD163^+^ are denoted as MoN and MoP, respectively. The expression pattern of these markers in the captured cells were consistent with that in the monocytes of the MoN population (Fig. 3c).

### 3.3 Outgrowth capacity of captured cells

**Fig. 4:**
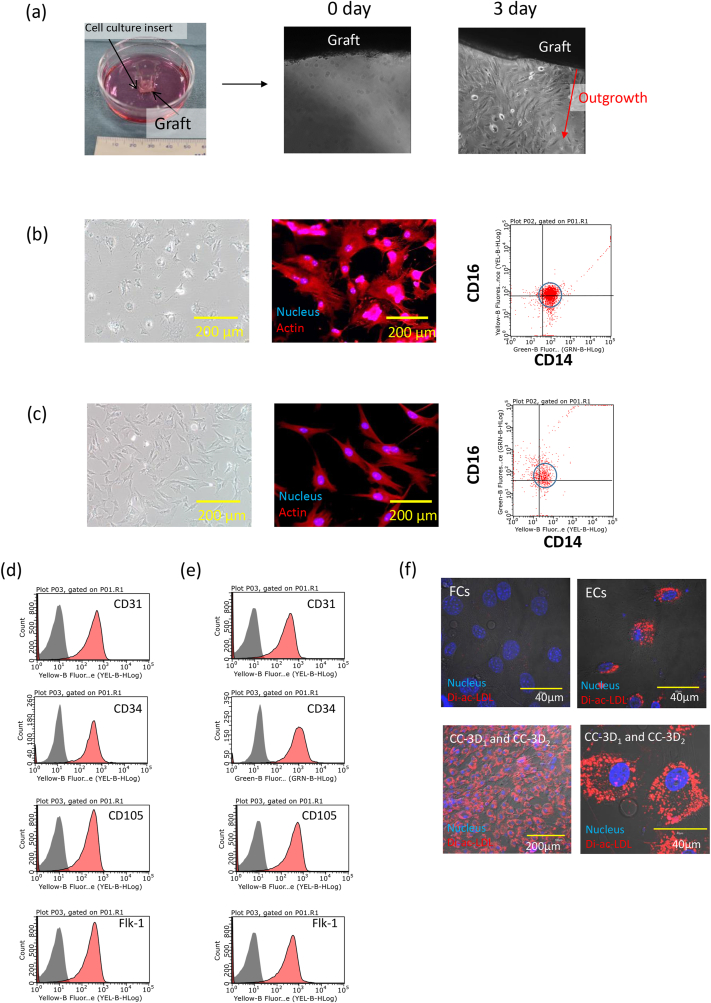
**Outgrowth capacity and surface marker analysis of captured cells a**, Outgrowth capacity was evaluated on the cell culture plate. The cells were grown from the tissue after 3 days. **b,c,** Two-type cells were isolated from the single colony cultivation. The cells were classified into (**b)** filopodia-shaped (CC-3D_1_) and **(c)** spindle-shaped (CC-3D_2_) cells based on the difference in morphology. These cells were expressed the surface maker of CD16/CD14. **d**,**e**, Surface marker expression of CD31, CD34, CD105, and Flk-1 on (**d**) CC-3D_1_ and (**e**) CC-3D_2_ were indicated. **f**, Di-ac-LDL uptake of the mixture of CC-3D_1_ and CC-3D_2_ was compared to the fibroblast and endothelial cells on CLSM observation.

The outgrowth capacity of the captured cells was also evaluated. When the transplanted graft tissues were incubated in a culture dish for 3 days, outgrowth cells were observed (Fig. 4a). When these cells were cultured from a single-cell colony, two cell types with specific morphological characteristics were isolated (Fig. 4b and c). Cells characterized by a filopodia morphology (CC-3D_1_) weakly expressed CD14 and CD16 (Fig. 4b), while cells characterized by a spindle shape (CC-3D_2_) also weakly expressed CD14 and CD16 (Fig. 4c). CD31, CD34, CD105, and Flk-1 expression was observed in CC-3D_1_ and CC-3D_2_ cells (Fig. 4d and e) and confirmed using cell staining (Supplemental Fig. S3). The uptake of Dil-ac-LDL by these cells was consistent with that by endothelial cells (Fig. 4f). These results indicate that the captured cells had cell growth capacity and CC-3D_1_ and CC-3D_2_ had similar surface markers as CC-3H and endothelial cells.

### 3.4 Gene expression analysis of CC-3H, CC-3D_1_, and CC-3D_2_

**Fig. 5:**
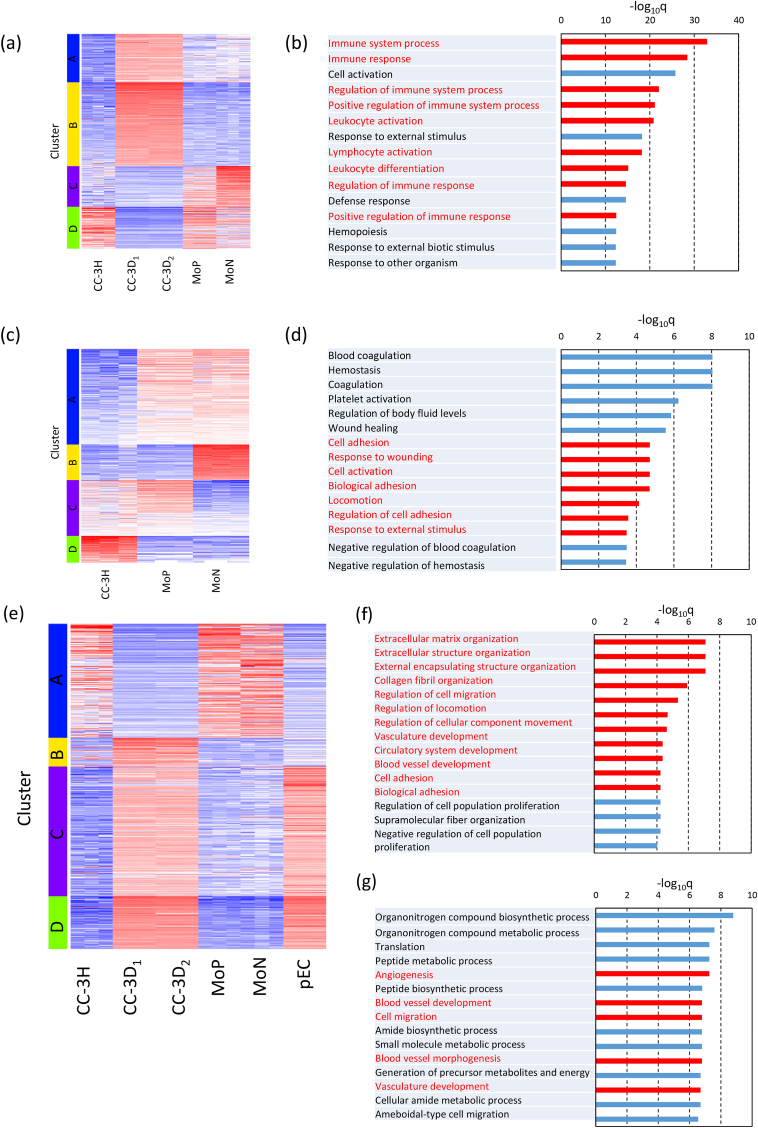
**RNA sequencing analysis. a**, Heat map representation of CC-3H, CC-3D_1_, CC-3D_2_, MoN, and MoP genes based on the K-means analysis with four clusters. **b**, Annotation analysis of selected genes in cluster D was summarized in the table. **c**, Heat map representation of k-means analysis of genes extracted in cluster c and d of the heat map. **d**, Annotation analysis of selected genes in cluster D was summarized. **e**, Heat map representation of CC-3H, CC-3D_1_, CC-3D_2_, MoN, MoP, and pEC genes based on the K-means analysis with four clusters. **f**, Annotation analysis of selected genes in cluster B groups was summarized. **g**, Annotation analysis of selected genes in cluster C and D groups was summarized. The data in each sample are expressed in three independent experiment results.

The cells captured on the graft expressed monocyte surface markers and showed outgrowth capacity. These cells might have acquired endothelial cell characteristics over 3 days. To evaluate global transcriptional changes in CC-3H, CC-3D_1_, and CC-3D_2_, RNA-sequencing analysis was performed (Fig. 5). The top 2000 ranked genes were divided into four clusters by k-means analysis (Fig. 5a). In cluster D, upregulation of immune-related genes was observed in CC-3H, MoN, and MoP (Fig. 5b). These genes were not upregulated in CC-3D_1_ and CC-3D_2_. The 689 genes that were specifically expressed in the monocyte populations and CC-3H were selected from clusters C and D as shown in Fig. 5a, and the expression levels between these cell populations were compared using k-means clustering. Gene expression was reanalyzed using non-hierarchical clustering (Fig. 5c). The genes in cluster D were adhesion related (Fig. 5d). Upregulation of cell adhesion-related genes was observed in CC-3H but not in MoN and MoP. These analyses suggest that immune- and adhesion-related gene upregulation in CC-3H cells is consistent with the immunohistochemical analysis and reveals the high affinity of the cells for integrin α4β1 ligand peptides.

Next, we compared the gene expression profiles between the captured cells and porcine endothelial cells (Fig. 5e). The genes in cluster B were upregulated only in CC-3D_1_ and CC-3D_2_. In the k-means analysis, cluster B were enriched in extracellular matrix-related gene expression (Fig. 5f). Clusters C and D were enriched in angiogenesis- and vascular development-related genes, which were upregulated in both pEC and CC-3D_1_ and CC-3D_2_ (Fig. 5g). These data indicate that CC-3H cells were unlikely to have differentiated into macrophages.

At inflammation and injury sites, monocytes differentiate into macrophages. To evaluate whether the captured cells were converted into macrophages, we checked the expression levels of macrophage- and monocyte-related genes (Supplemental Fig. S4). The monocyte subpopulation MoP had both monocyte and macrophage characteristics; however, MoN and CC-3H mainly expressed monocyte-related genes. CC-3D_1_ and CC-3D_2_ cells lost both characteristics when cultured for 3 days. These data suggest that efficient differentiation of monocytes into endothelial-like cells on the REDV-modified surface could occur via integrin activation signals.

### 3.5 Tube formation assay

**Fig. 6:**
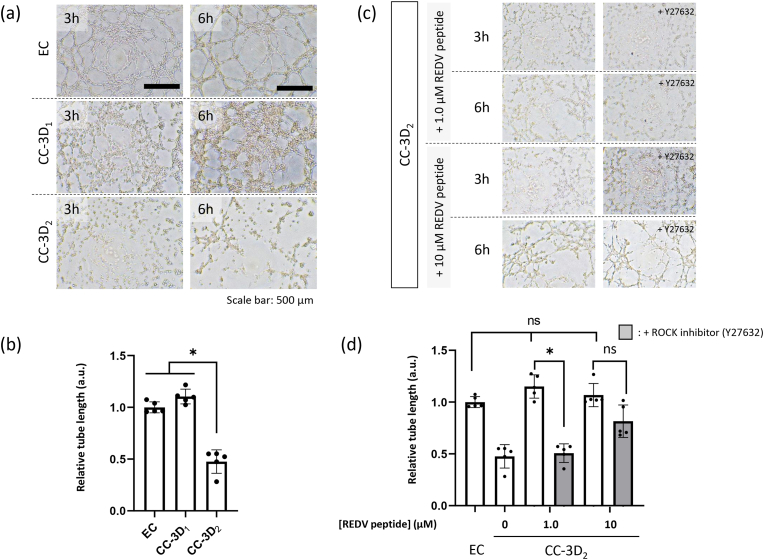
**Tube formation assay a**, Tube formation of EC, CC-3D_1_ and CC-3D_2_ were observed at 3 and 6 h after incubation. **b**, The tube length of EC, CC-3D_1_ and CC-3D_2_ were quantitatively evaluated by using Angiogenesis analyzer on ImageJ software. **c**, To investigate the impact of integrin binding peptide on tube formation, CC-3D_2_ was cultured with the REDV peptide as the stimulator for integrin α4β1. The Y27632 was used as the inhibitor for the ROCK signal. **d**, Tube length of EC and CC-3D_2_ with and without the REDV peptide and Y27632 were quantitatively evaluated by using Angiogenesis analyzer on ImageJ software. Data are indicated as the means ± standard deviation (n = 5, *:p < 0.05, n.s.: Not significant).

To evaluate the endothelial function of the captured cells, in vitro microtube formation was evaluated using a Matrigel plate (Fig. 6). A tube structure similar to that of ECs was formed in the case of CC-3D_1_, whereas the tube was partially formed in the case of CC-3D_2_ (Fig. 6a). The quantitative data suggested that the tube length of CC-3D_2_ was approximately half that of ECs and CC-3D_1_ (Fig. 6b). Based on the mRNA-sequencing analysis, CC-3D_2_ was cultured on the Matrigel plate in the presence of the REDV peptide, which was used as the integrin-binding peptide (Fig. 6c). As a result, the CC-3D_2_ formed a microtube structure comparable to that of ECs when the cells were cultured with the peptide at concentrations of 1.0 and 10 μM. This peptide was added to the medium as a free peptide. This result indicates that tube formation was sufficiently induced with 1 μM of the REDV peptide. On the contrary, tube formation was inhibited by adding a Rho-associated protein kinase inhibitor (Y-27632) which is known to inhibit Rho/ROCK signaling related to important mediators in a number of angiogenic processes [27]. The tube length is shown quantitatively in Fig. 6d. Y-27632 inhibited the tube formation of CC-3D_2_ compared with EC when the cells were cultured with the peptide at a concentration of 1.0 μM. In contrast, tube formation was not inhibited by the addition of Y-27632 when the cells were cultured with the peptide at a concentration of 10 μM. This result indicated that the excess peptide signaling promoted tube formation even under the addition of ROCK inhibitors. These results suggest that captured cells exhibited tube formation activity as an endothelial function, which was strongly promoted by the integrin-binding peptide.

### 3.6 In vitro direct differentiation of monocyte to endothelial phenotype

**Fig. 7:**
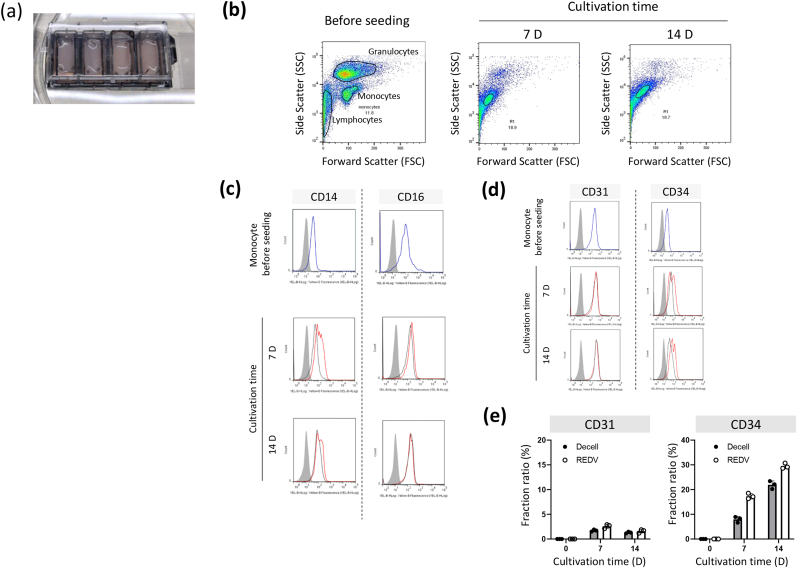
**In vitro differentiation assay on decellularized graft a**, Schematic image of the culture plate with decellularized tissue and REDV-modified decellularized tissue. **b**, Two-dimensional plot showing the SSC and FSC signals of pre-seeding cells and cultured cells on the REDV-modified surface for 7 and 14 days. **c, d**, Surface marker analysis by FACS; the surface maker expressions of CD14 and CD16 are indicated. The blue line indicates the expression of the monocyte fraction. The black and red lines indicate the expression levels of the cells captured on the Un-graft and P-graft surfaces, respectively. **d**, Surface marker expression of CD31 and CD34 were indicated. In both cases, monocytes and cultured cells on decellularized and REDV-tissue surface for 7 and 14 days were evaluated. The gray-filled distribution indicates the unstained cells. The blue line indicates the antibody-stained monocyte. Black and red lines indicate the antibody-stained cells cultured on the unmodified and REDV-modified decellularized surfaces, respectively. **e**, Fraction rate of CD31 and CD34 in the cultured cells on unmodified and REDV-modified surfaces plotted against cultivation time. (For interpretation of the references to color in this figure legend, the reader is referred to the Web version of this article.)

Furthermore, we investigated the direct in vitro differentiation of monocytes on P-grafts. Leukocytes isolated from porcine blood were seeded on the graft surface (Fig. 7a) and cultured for 7 and 14 days. Leukocytes had three main fractions in the two-dimensional analysis with forward scatter (FSC) and side scatter (SSC). After cultivation for 7 and 14 days, adherent cells were shown to mainly contain a monocyte population (Fig. 7b). This indicated that a monocyte fraction adhered to the luminal surface of the P- and Un-grafts. The cells expressed the surface markers CD14 and CD16. The expression level of CD14 on the REDV-modified surface was slightly higher than that on the unmodified surface (Fig. 7c). The CD34 expression of cells adhered to the REDV surface increased with cultivation time, while CD31 expression did not (Fig. 7d and e). These results indicate that blood-circulating monocytes adhered to the decellularized surface and adherent cells were differentiated by immobilized REDV peptide on the decellularized surface.

### 3.7 In vivo vascular tissue regeneration

**Fig. 8:**
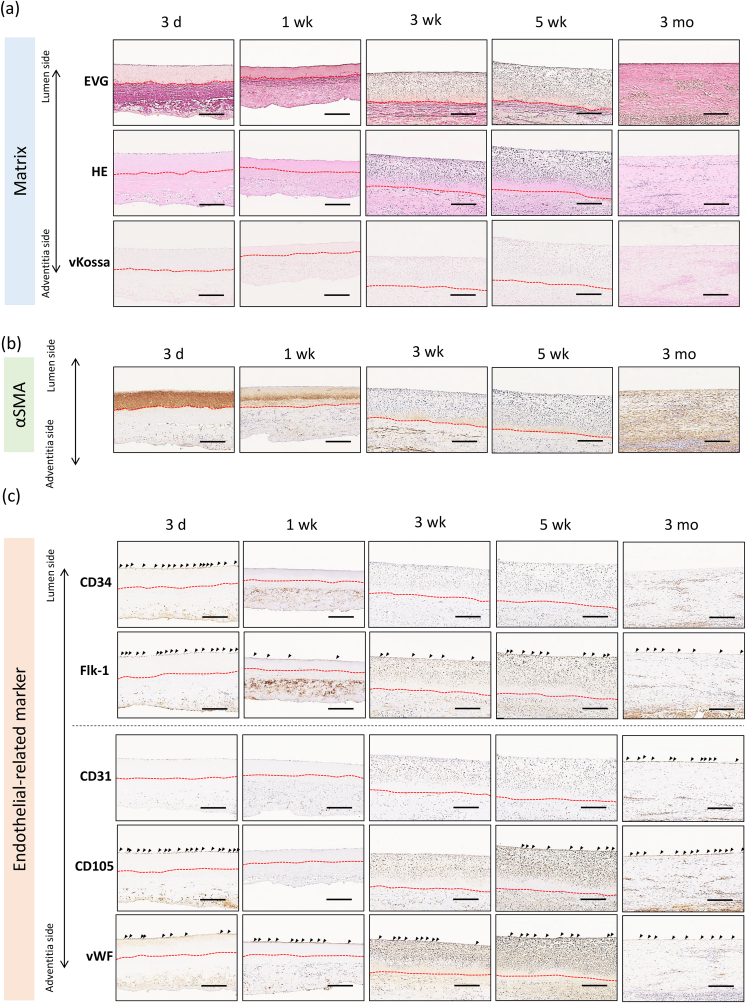
**Tissue regeneration process of the decellularized vascular graft evaluated by immunohistostaining for 3-month graft transplantation. a**, Longitudinal cross-section of the graft was evaluated by HE, EVG, and von Kossa staining to investigate the matrix structure and degradation process. **b**, Longitudinal cross-section of the graft was evaluated by αSMA staining to investigate the reconstruction of medium layer. **c**, Longitudinal cross-section of the graft was evaluated by atniCD31, antiCD34, antiCD105, and antiVEGFR2 antibodies staining to investigate the reconstruction of endothelial layer. Scale bars indicate 200 μm. Red dotted-lines indicate the border between the medium layer and external elastic lamina. Cells expressing antigens on their surface of the graft are indicated with arrows. (For interpretation of the references to color in this figure legend, the reader is referred to the Web version of this article.)

The tissue regeneration process by the cells captured for 3 months was evaluated by histological analysis (Fig. 8).

#### 3.7.1 Matrix component

The EVG-stained histological images indicated that the internal and external elastic lamina structures of the graft had clearly developed at 3 days (Fig. 8a). The external elastic lamina persisted for 5 weeks. The HE-stained histological images indicated that the captured cells on the luminal surface of the graft were present in layers for up to 1 week after transplantation. Three weeks after transplantation, the captured cells proliferated and infiltrated the medium layer, rendering the medium layer thicker. Five weeks after transplantation, matrix components increased with decreasing cell density. After 3 months, the cell density was largely decreased and an extracellular matrix was formed. von Kossa-stained histological images indicated that calcium had not been deposited during the 3 months. The thickness of the tunica media of the graft increased by approximately two-fold as the cells infiltrated the graft wall.

#### 3.7.2 αSMA

The tunica media of the graft expressed αSMA, which was derived from an ostrich matrix (Fig. 8b). After cell infiltration into the medium layer at 3 and 5 weeks, the matrix was not stained with the anti-αSMA antibody. These results suggest that the graft matrix gradually degraded with cell infiltration. After 3 months, the infiltrated cells expressed αSMA in the medium layer, which was derived from the host cells.

#### 3.7.3 Endothelial-related marker

CD34 and Flk-1 expression was evaluated as progenitor markers (Fig. 8c). The cells captured on the luminal surface after 3 days expressed CD34 and Flk-1. Thereafter, Flk-1 expression was maintained during the cell infiltration process at 3 and 5 weeks, whereas CD34 expression gradually decreased with time. At 3 months, the luminal layer expressed Flk-1 but not CD34.

Next, the expression of endothelial markers CD31, CD105, and vWF was evaluated. The captured cells expressed CD105 but not CD31. At three months, the luminal layer expressed CD31 and CD105. In contrast, after 3 months, the cells expressed vWF. Finally, the expression of CD31, CD105, and vWF in the luminal layer was similar to that in the native endothelial layer.

When the graft was transplanted into a goat median artery for 1 year, luminal layers were formed by the expression of vWF and the cells in the medium layer as indicated by αSMA were similar to that in the 3-month transplantation porcine model (Supplemental Fig. S5). These results indicated that captured cells migrated to the media layer and reconstructed not only the intima but also the tunica media layer. In addition, a vascular-like structure was completed approximately 3 months after transplantation.

## 4 Discussion

The initial response when blood flow is provided to graft material is thrombosis formation. Originally, we revealed that the REDV peptide suppresses platelet adhesion and fibrin clot deposition in a peptide-sequence-specific manner based on the REDV-peptide density-controlled acellular surface with silane coupling agents [20,22]. In the case of peptide modification with POG7G3-REDV, microthrombosis formation on the graft surface was effectively suppressed [28] (Fig. 2). Recently, the anti-thrombosis and anti-platelet adhesion properties of the REDV-peptide modification have been discussed [29,30]. In these reports, it was pointed out that the hydrophilicity of the REDV peptide was involved in the suppression of fibrinogen absorption [30]. Anti-thrombogenic properties of the CAG and YIGSR peptides have also been reported [31,32]. Moreover, hydrophilic polymers, polypeptide [33], and heparin-coatings have been investigated as anti-thrombogenic surfaces [34]. These reports suggest that hydrophilicity plays a major role in suppression [29]. In general, surface-free energy is related to protein absorption on the polymer surface, and it has been proven that fibrinogen absorption increases with the reduction in surface-free energy [35]. That is, surface hydrophilicity is thought to be related to the suppression of microthrombosis formation. Additionally, the shielding effect against the RGD peptide sequence in the decellularized matrix is another factor in the suppression of fibrinogen binding because fibrinogen binds through the RGD sequence of the extracellular matrix [32]. Suppression of thrombosis formation on the peptide-modified graft surface is an important process for neointimal formation and tissue regeneration.

Ligand immobilization for blood vessels and stents is an attractive strategy for promoting endothelialization, and it is expected to suppress thrombus formation and intimal thickening over long-term periods. Anti-CD34 antibody-immobilized stents have been developed [36] and used in clinical situations. Artificial blood vessels modified with DNA aptamers and REDV peptides, the ligands of which are used for rapid endothelialization, have also been reported [[37], [38], [39]]. Wei reported that REDV-modified stents induced rapid endothelialization in vivo [40]. This modification promotes the proliferation of surrounding endothelial cells and suppresses intimal thickening. In the case of stents, endothelialization is accomplished by the surrounding residential stem/progenitor cells because the stent strut is very thin. Moreover, the vascular tissue is repaired by the proliferation of surrounding endothelial cells in cases of short intimal defects [15]. In many vascular graft studies, graft patency and endothelialization have been investigated in mice or rats using a graft of less than 10 mm in length [41]. In this situation, endothelialization may be accomplished through endothelial cell growth from the anastomotic site. In a realistic situation, an artificial vascular graft should be more than 5 cm in length, and endothelialization of the entire luminal surface by residential stem cells from the anastomotic site likely does not occur. Therefore, it is very important to verify the tissue regeneration of TEVGs using an appropriate graft size in a large animal model.

Here, we demonstrated the vascular tissue regeneration ability of blood-circulating cells captured on a REDV-modified acellular surface using a long-bypass graft in a minipig transplantation model. Our data indicated that the luminal surface of the graft was rapidly covered with circulating blood cells after 1 day (Fig. 2c) and the cells expressed the surface marker, which was consistent with the monocyte subpopulation (Fig. 3b and c). RNA sequencing analysis also demonstrated that the captured cells expressed immune-related genes similar to monocytes (Fig. 5) and indicated that the captured cells were a monocyte subpopulation.

Moreover, the captured cells may have originated from macrophages. In 2020, Smith Jr. et al. suggested that blood-circulating monocytes differentiate into EC and macrophages in the vascular remodeling process [17]. However, in our case, RNA-sequencing data suggested that the captured cells did not express macrophage-associated genes during differentiation (Supplemental Fig. S4). The captured monocyte at 3 h already expressed Flk-1, which is known as the EC maker. This could be related to the efficient differentiation of monocytes into EC-like cells on the REDV-modified surface via integrin activation signals. Caidao et al. reported that integrin α4β1 plays a key role in regulating endothelial cell formation [42]. Furthermore, the collagen matrix has been reported to be suitable for angiogenesis formation [43]. Integrin α4β1 initiates blood vessel maturation [44], and a previous study suggested that integrin signal activation is involved in EC differentiation and angiogenesis. These findings provide supportive evidence for our findings. We demonstrated that the differentiation of monocytes into EC-like cells was also supported by the tube formation assay and surface marker changes in the in vitro experiments (Fig. 6, Fig. 7). However, the differentiation efficiency appeared to be considerably lower than that observed in the in vivo study. One of the limitations of this in vitro experiments is that the cultured cells were not the same cell population as that of the recruited cells. Additionally, under in vivo environments where tissue regeneration occurs, dynamic shear stress by blood flow and various growth factors from the surrounding niche are involved in cell proliferation and differentiation. Therefore, the in vitro differentiation efficiency might not be perfectly reflected when compared with in vivo conditions.

Presumably, monocyte differentiation was efficiently induced in a niche environment triggered by integrin activation signaling. Notably, using time-resolved tissue-staining images, we successfully demonstrated that the captured cells infiltrated from the lumen side to the medium layer of the graft and were involved in reconstructing not only the endothelium but also the tunica media layer (Fig. 8). It is widely known that muscle cells have different embryonic origins from other cells [45]. Yamashita et al. suggested that Flk-1 positive cells derived from ES cells have the ability to differentiate into both endothelial and smooth muscle cell lines for vascular tissue reconstruction [46]. Simper et al. suggested that the circulating smooth muscle progenitor cells originated in the mononuclear cell fraction and showed that these cells were characterized by integrin expression [47]. Despite these results, the origin of SMCs for reconstructing vascular tissues in the adult body has not been clearly identified. Our experiments showed that monocytes infiltrated the medium layer of the graft and some of them expressed aSMA-positive cells (Fig. 8). These cells clearly showed a phenotype different from that of cells on the endothelium. The captured cells were Flk-1 positive, which is consistent with previously reported phenotypes [47]. In addition, the activation of integrin signaling was similar to that in previous reports. Although the differentiation mechanisms have not been elucidated, we hypothesized that the monocyte subpopulation reconstructs the endothelium and vascular wall via integrin signal activation.

Asahara reported that EPCs express CD34 and Flk-1 [1] and that EPC transplantation improves neovascularization in an ischemia model [4,48]. Cardiovascular progenitor cells were isolated using the Flk-1 marker [49]; therefore, the surface marker of Flk-1 is important as a marker for cells to regenerate blood vessel [50]. In this study, captured cells expressed CD34 and Flk-1 and the surface markers were similar to those reported for progenitor cells. However, a number of studies have indicated that EPCs are not a single cell population, and a consensus has not been reached on the existence of circulating EPCs [5]. ECFCs have angiogenesis ability and are resident cells in many organs. However, the origin of ECFCs is not well understood, and they still represent a heterogeneous population [9]. Although evidence has not been obtained showing that mouse circulating ECFCs are recruited to an injured site, a number of studies of human ECFCs have shown that circulating ECFCs play a role in health and disease [51]. Although it is possible to discuss the possibility of endothelial-like cells based on surface markers, cell species cannot be defined using only marker expression under the current knowledge. Therefore, we conducted a tube formation assay to evaluate the cells’ angiogenesis ability. Tube formation by endothelial cells is important for angiogenesis and neointimal formation. The results of the assay clearly indicated tube formation by CC-3D_1_(Fig. 6). Notably, CC-3D_2_ presented a spindle shape and formed a tube with low activity, while CC-3D_1_ showed a filopodia morphology. Filopodia have been reported to be involved in a number of cellular processes, including wound healing [52]. In an in vivo study, filopodia were formed during angiogenesis process [53]. Thus, the angiogenic potential of proliferating and filopodia-presenting cells was consistent.

Several studies have reported the contribution of monocytes to vascular regeneration on artificial vascular grafts. In 2010, Roh et al. reported that the recruitment of monocytes and inflammatory processes were involved in the regenerative phase of the cell-free vascular graft [54]. Koobatin et al. demonstrated the endothelialization, remodeling, and development of the vascular function of VEGF-modified cell-free grafts in the ovine transplantation model in 2016 [55]. Furthermore, Smith et al. reported that circulating monocytes directly promoted vascular regeneration on cell-free VEGF-modified grafts, with monocytes differentiating into EC via macrophages and EC-like phenotypes [17]. Using in vitro and mouse models, Nasiri et al. indicated that the recruitment of monocytes and macrophages contributes to the revascularization of cell-free grafts [56]. These previous findings do not contradict the experimental results obtained in our study but instead support the context of our findings. Considering both these data and our findings, we may affirm that monocytes strongly contribute to vascular tissue regeneration. The large-animal experimental results with multiple early time points provided in our data are clear evidence to revealing that vascular tissue can be reconstructed by monocytes. In particular, CC-3D_1_ cells represent a monocyte subpopulation that is strongly involved in vascular regeneration. Unfortunately, this study is limited by the detailed molecular mechanisms by which the REDV peptide promotes vascular regeneration through captured cells not being fully elucidated. However, we consider that this will be an important finding for future research which should elucidate the mechanism of vascular reconstruction in the adult body. In future work, we plan to investigate the molecular mechanisms associated with the differentiation from monocyte to vascular endothelial and smooth muscle cells using a single-cell sequencing analysis. In addition, the identification of captured cells and spatiotemporal proof involved in graft patency and tissue regeneration would be major evidence of the effectiveness of blood vessel application in clinical sites. These findings could also be useful in evidence-based medicine.

## 5 Conclusion

This study revealed that blood-circulating monocyte subpopulations have proliferative and reconstruction abilities of vascular tissue. Moreover, this work provides multiple time-point evidence that vascular tissues can be reconstituted by monocytes in an adult host body.

## Statement of significance

Although the clinical application of cell-free tissue-engineered vascular grafts (TEVGs) has been proposed, vascular tissue regeneration mechanisms have not been fully clarified. Recently, several reports have provided the contribution of monocytes to vascular regeneration on artificial vascular grafts. In those reports, the monocyte would differentiate into EC via macrophage and EC-like phenotype. Here, we investigated vascular tissue regeneration with both early time points and long-term periods by using REDV-modified acellular graft of clinically useful size. Here, we proved that monocyte subpopulations are directly involved in vascular remodeling not only the endothelium but also the medium layer of the vascular tissue based on the multi-time point observation in large animal model. Moreover, the integrin activation by the REDV peptide contributed to the monocyte differentiation using the total RNA sequencing analysis. This will be an important finding for future studies to elucidate the mechanism of vascular reconstruction in the adult body.

## CRediT authorship contribution statement

**Atsushi Mahara:** Conceptualization, Project administration, Investigation, Methodology, Validation, Writing – original draft, Writing – review & editing. **Manabu Shirai:** Methodology, Validation, Data curation, Visualization. **Raghav Soni:** Investigation, Validation, Data curation. **Hue Thi Le:** Investigation, Methodology. **Kaito Shimizu:** Investigation. **Yoshiaki Hirano:** Supervision. **Tetsuji Yamaoka:** Conceptualization, Project administration, Funding acquisition, Validation, Writing – review & editing, Supervision.

## Declaration of competing interest

The authors declare that they have no known competing financial interests or personal relationships that could have appeared to influence the work reported in this paper.

## Data Availability

Data will be made available on request.
