# Supplementary material for: Vascular tissue reconstruction by monocyte subpopulations on small-diameter acellular grafts via integrin activation
Source: Mater Today Bio. 2023 Oct 28;23:100847. doi: 10.1016/j.mtbio.2023.100847 (PMC10632538; doi:10.1016/j.mtbio.2023.100847)
Supplement: Multimedia component 1 [file mmc1.pptx]

## Slide 1
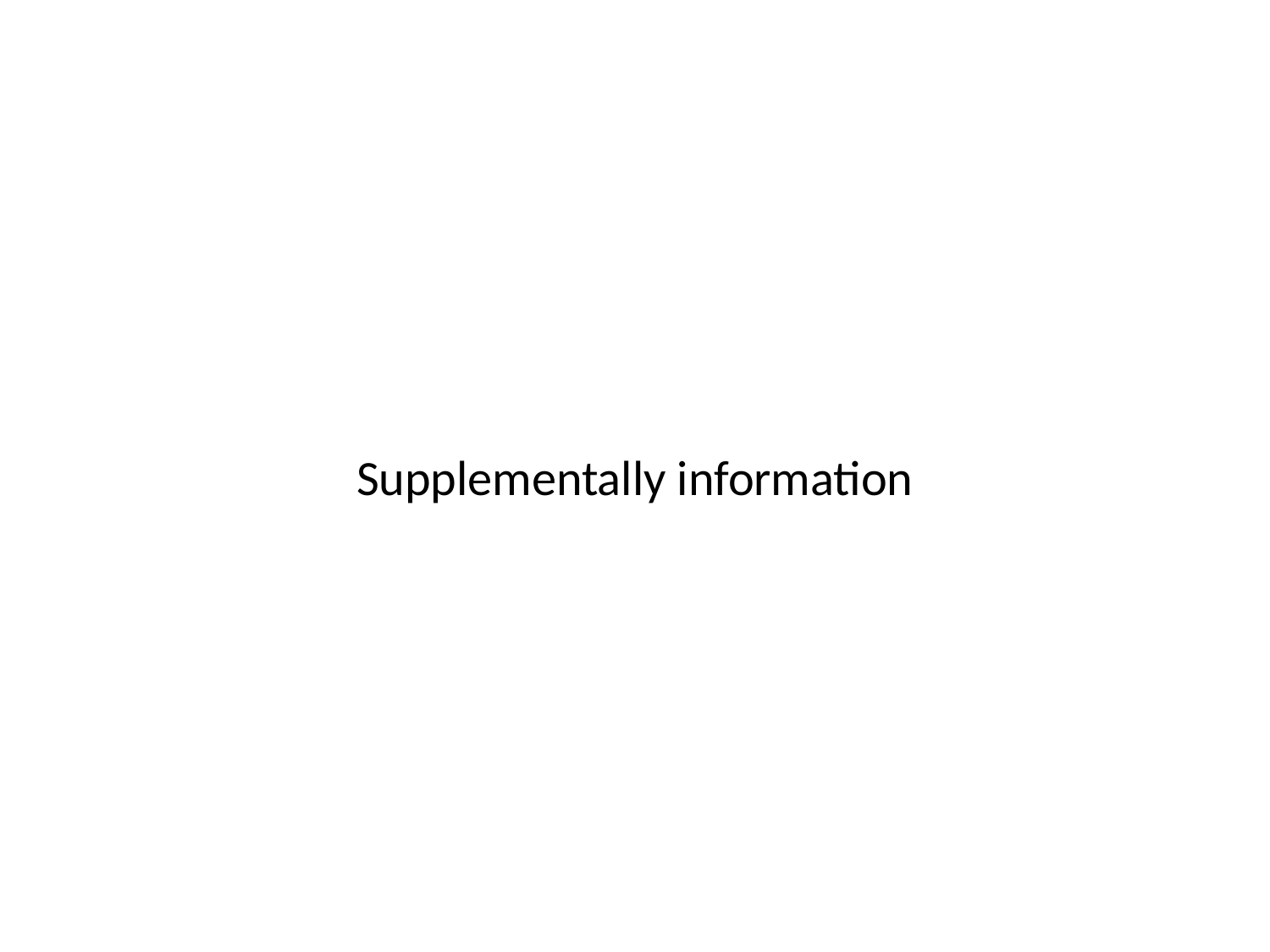

Supplementally information

## Slide 2
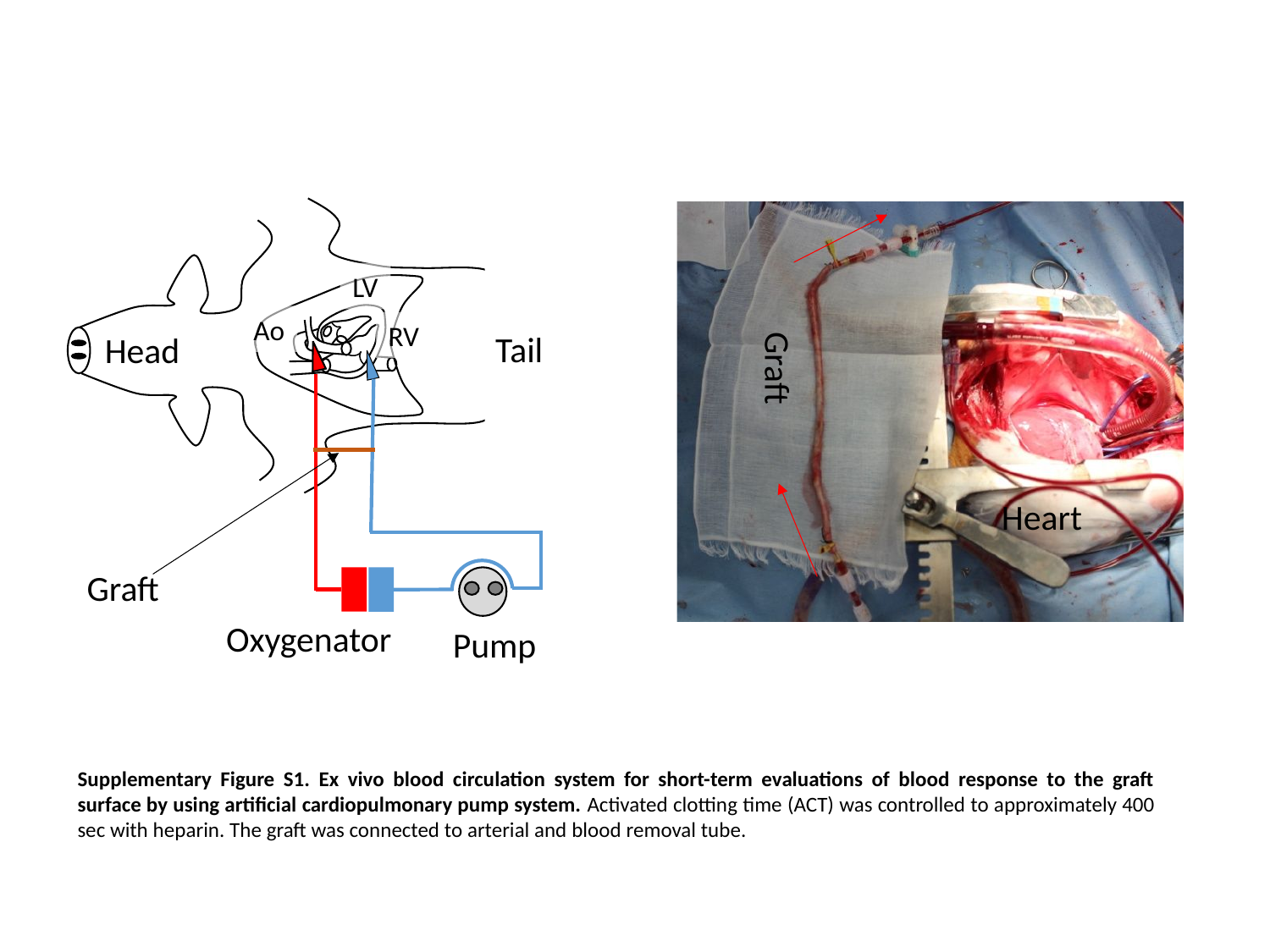

LV
Ao
RV
Tail
Head
Graft
Oxygenator
Pump
Graft
Heart
Supplementary Figure S1. Ex vivo blood circulation system for short-term evaluations of blood response to the graft surface by using artificial cardiopulmonary pump system. Activated clotting time (ACT) was controlled to approximately 400 sec with heparin. The graft was connected to arterial and blood removal tube.

## Slide 3
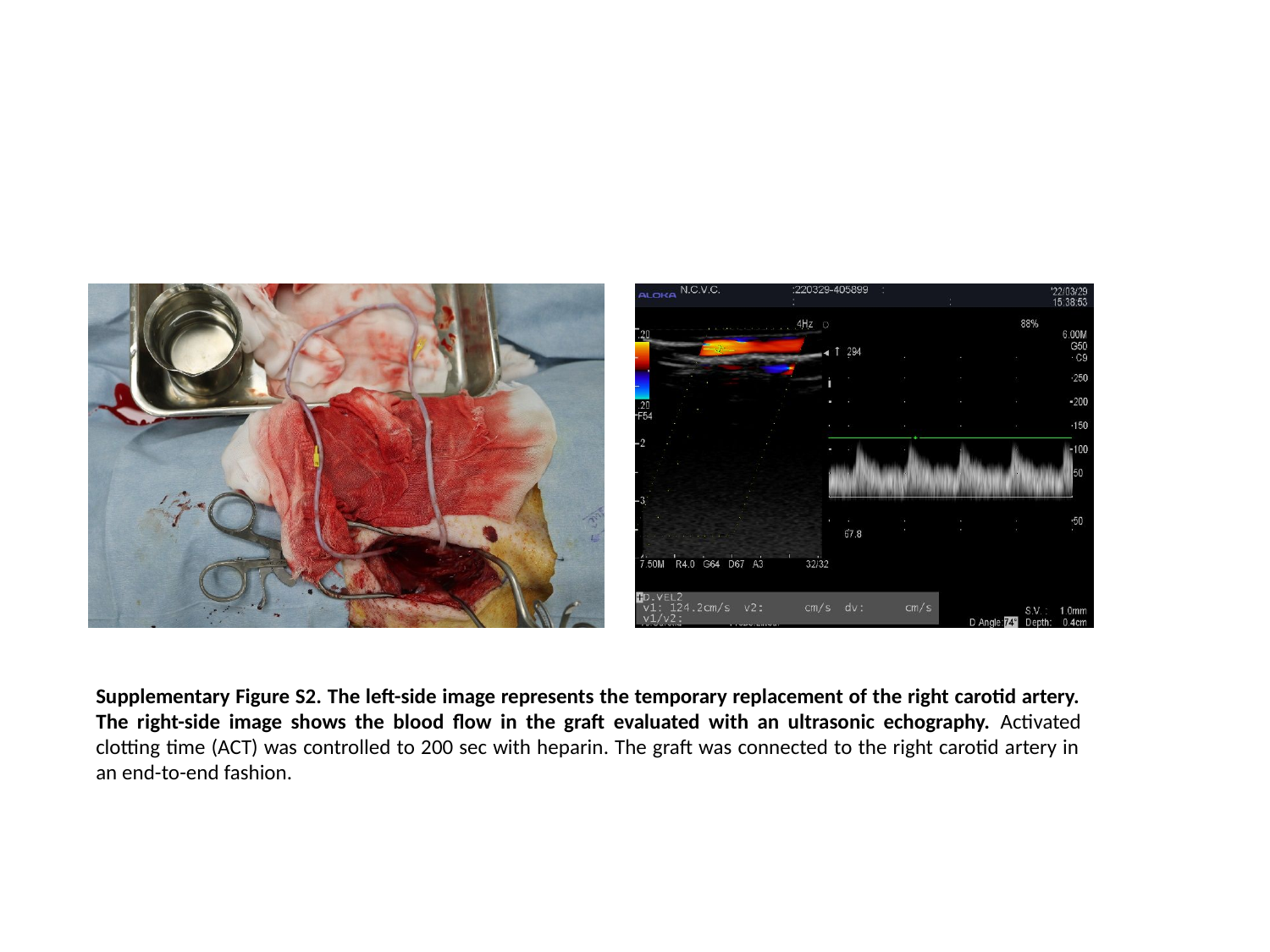

Supplementary Figure S2. The left-side image represents the temporary replacement of the right carotid artery. The right-side image shows the blood flow in the graft evaluated with an ultrasonic echography. Activated clotting time (ACT) was controlled to 200 sec with heparin. The graft was connected to the right carotid artery in an end-to-end fashion.

## Slide 4
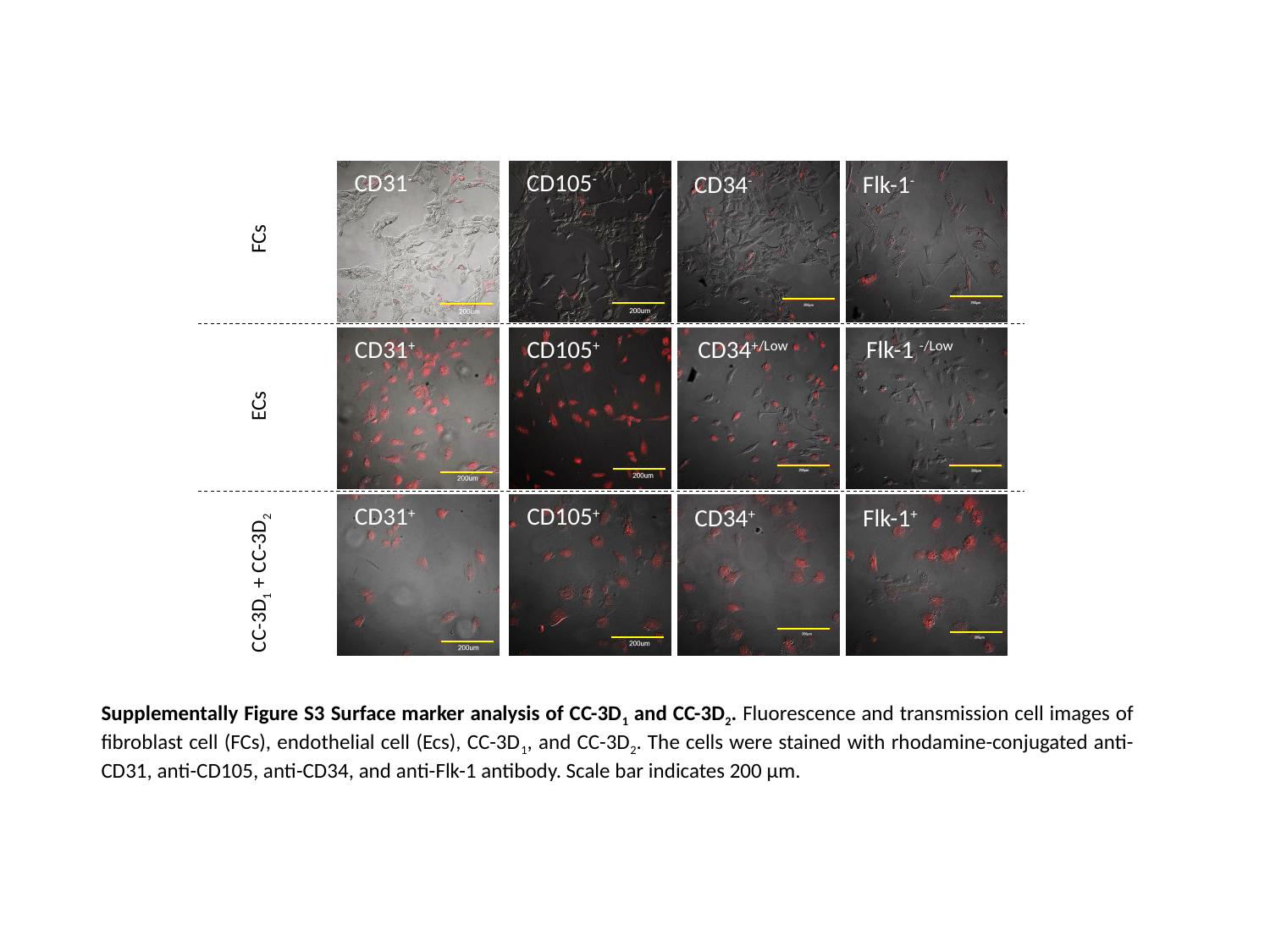

CD31-
CD105-
CD34-
Flk-1-
CD31+
CD105+
CD34+/Low
Flk-1 -/Low
CD31+
CD105+
CD34+
Flk-1+
FCs
ECs
CC-3D1 + CC-3D2
Supplementally Figure S3 Surface marker analysis of CC-3D1 and CC-3D2. Fluorescence and transmission cell images of fibroblast cell (FCs), endothelial cell (Ecs), CC-3D1, and CC-3D2. The cells were stained with rhodamine-conjugated anti-CD31, anti-CD105, anti-CD34, and anti-Flk-1 antibody. Scale bar indicates 200 μm.

## Slide 5
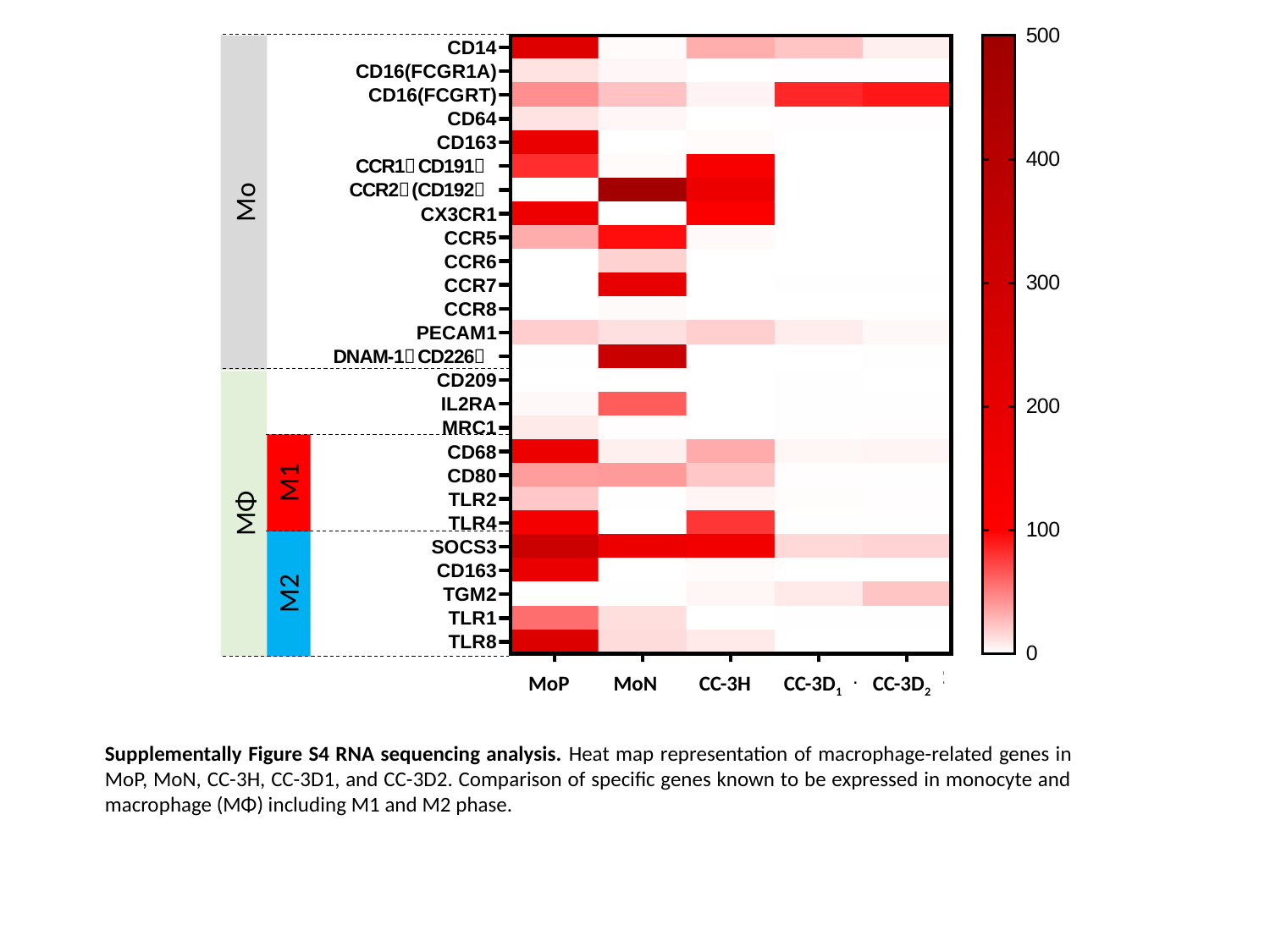

Mo
M1
MΦ
M2
MoP
MoN
CC-3H
CC-3D1
CC-3D2
Supplementally Figure S4 RNA sequencing analysis. Heat map representation of macrophage-related genes in MoP, MoN, CC-3H, CC-3D1, and CC-3D2. Comparison of specific genes known to be expressed in monocyte and macrophage (MΦ) including M1 and M2 phase.

## Slide 6
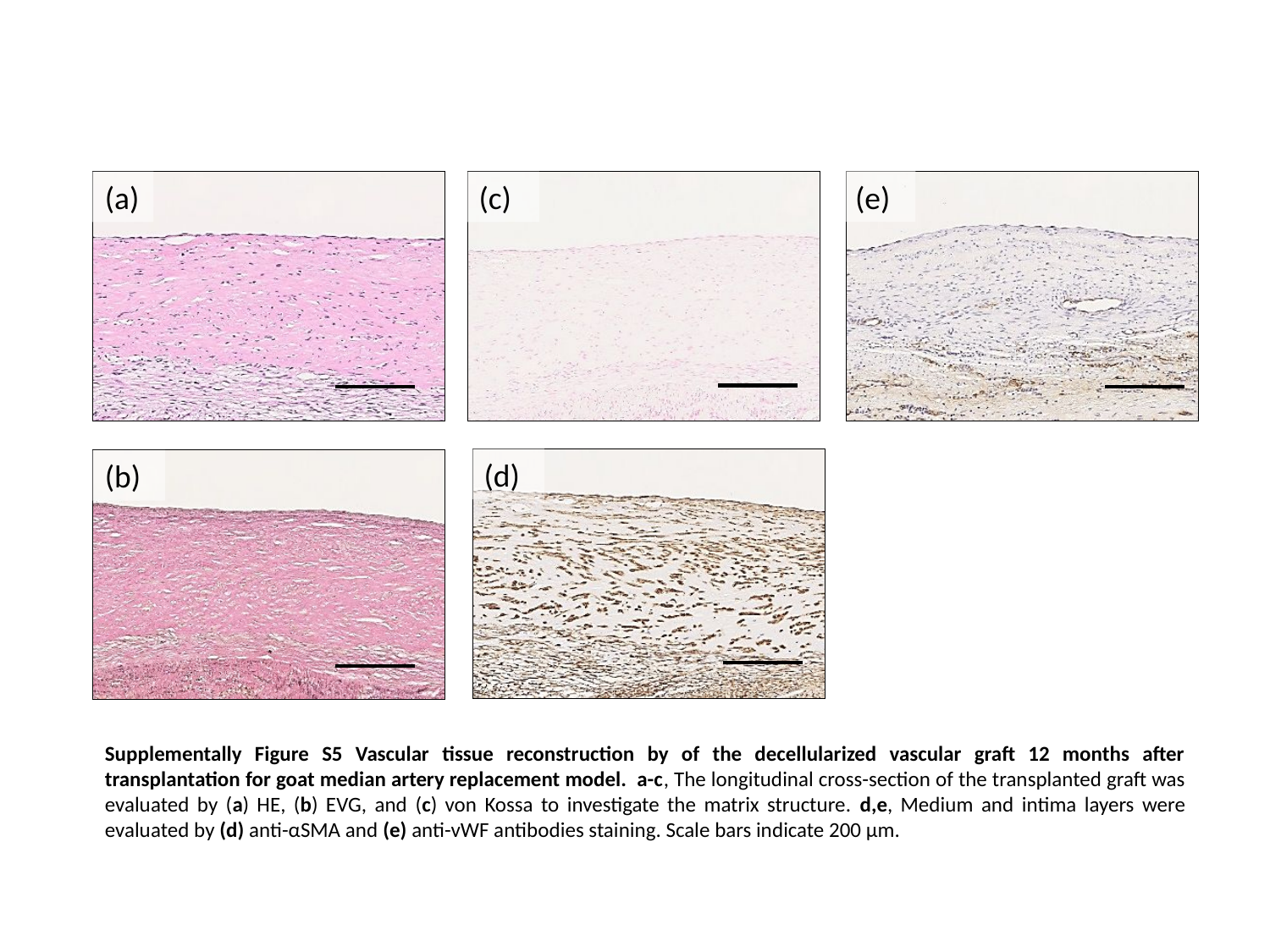

(e)
(a)
(c)
(d)
(b)
Supplementally Figure S5 Vascular tissue reconstruction by of the decellularized vascular graft 12 months after transplantation for goat median artery replacement model. a-c, The longitudinal cross-section of the transplanted graft was evaluated by (a) HE, (b) EVG, and (c) von Kossa to investigate the matrix structure. d,e, Medium and intima layers were evaluated by (d) anti-αSMA and (e) anti-vWF antibodies staining. Scale bars indicate 200 μm.
